# Effects of ambient carbon monoxide on daily hospitalizations for cardiovascular disease: a time-stratified case-crossover study of 460,938 cases in Beijing, China from 2013 to 2017

**DOI:** 10.1186/s12940-018-0429-3

**Published:** 2018-11-26

**Authors:** Haibin Li, Jingwei Wu, Anxin Wang, Xia Li, Songxi Chen, Tianqi Wang, Endawoke Amsalu, Qi Gao, Yanxia Luo, Xinghua Yang, Wei Wang, Jin Guo, Yuming Guo, Xiuhua Guo

**Affiliations:** 1 Department of Epidemiology and Health Statistics, School of Public Health, Capital Medical University, Beijing, China; 2 Beijing Municipal Key Laboratory of Clinical Epidemiology, Capital Medical University, Beijing, China; 3 Department of Epidemiology and Biostatistics, College of Public Health, Temple University, Philadelphia, USA; 4 Department of Neurosurgery, Beijing Tiantan Hospital, Capital Medical University, Beijing, China; 5 Department of Mathematics and Statistics, La Trobe University, Melbourne, VIC Australia; 6 School of Mathematical Sciences and Center for Statistical Science, Peking University, Beijing, China; 7 Beijing Municipal Commission of Health and Family Planning Information Center, Beijing, China; 8 Global Health and Genomics, School of Medical Sciences and Health, Edith Cowan University, Perth, WA Australia; 9 Guanghua Group Pty Ltd, Melbourne, VIC Australia; 10 Department of Epidemiology and Preventive Medicine, School of Public Health and Preventive Medicine, Monash University, Melbourne, VIC Australia

**Keywords:** Carbon monoxide, Hospitalizations, Cardiovascular disease

## Abstract

**Background:**

Evidence focused on exposure to ambient carbon monoxide (CO) and the risk of hospitalizations for cardiovascular diseases (CVD) is lacking in developing countries. This study aimed to examine the effect of CO exposure on hospitalizations for CVD in Beijing, China.

**Methods:**

A total of 460,938 hospitalizations for cardiovascular diseases were obtained from electronic hospitalization summary reports from 2013 to 2017. A time-stratified case-crossover design was conducted to investigate the association between CO exposure and hospitalizations for total and cause-specific CVD, including coronary heart disease (CHD), atrial fibrillation (AF), and heart failure (HF). Stratified analysis was also conducted by age group (18–64 years and ≥ 65 years) and sex.

**Results:**

Linear exposure-response curves for the association between ambient CO exposure and hospitalizations for CVD was observed. Ambient CO was positively associated with hospitalizations for total CVD and CHD. However, the observed increased risk was not statistically significant for hospitalizations for AF and HF. The strongest effect of CO concentration was observed on the current- and previous-day of exposure (lag _0–1_ day). For a 1 mg/m^3^ increase in a 2-day moving average CO concentration, an increase of 2.8% [95% confidence interval (CI): 2.2 to 3.3%] and 3.0% (95% CI: 2.4 to 3.6%) in daily hospital admissions for CVD and CHD were estimated, respectively. This association was robust after adjusting for other copollutants and did not vary by age group and sex.

**Conclusions:**

Ambient CO exposure increased the risk of hospitalizations for CVD, especially for CHD in Beijing. Further studies are warranted to explore the association between ambient CO and hospitalizations for AF and HF.

## Introduction

Cardiovascular disease (CVD) is a leading cause of morbidity and mortality worldwide [1]. In China, approximately 29.8 million deaths in rural areas and 26.5 million deaths in urban areas were caused by CVD in 2015 [2]. Meta-analysis and epidemiological studies have reported that air pollution increased the risk of morbidity and mortality due to CVD [3–7]. Recently, Liu and colleagues conducted a nationwide time-series analysis in 272 major cities in China and found that ambient carbon monoxide (CO) exposure caused a 1.1% (95% confidence interval: 0.4 to 1.8%) increase in the risk of cardiovascular disease mortality per 1 mg/m^3^ increment [7]. However, the adverse effects of ambient CO on hospital admissions for cardiovascular disease has not been well described and the results were inconsistent across studies [7–12]. For example, *Koken,* et al. reported a significant association between short-term exposure to ambient CO and hospitalizations for CVD among elderly individuals in Denver [8]. However, this association between ambient CO and hospitalizations for CVD outcomes was found to be statistically insignificant by the Myocardial Ischemia National Audit Project [12].

In many large cities such as Beijing, the capital of China, traffic-related pollution has become a serious environmental problem. The total number of motor vehicles on the road in Beijing was nearly 5.64 million in 2017. Approximately 80% of ambient CO was attributed to exhaust emissions from motor vehicles. Ambient CO is a colorless, odorless and nonirritating gas pollutant. The adverse health effects of ambient CO exposure on cardiovascular mortality has been reported in multi-city analyses in China [7, 13]. To the best of our knowledge, only one multi-city study conducted in China examined the short-term effects of ambient CO on CVD hospital admissions [14]. This study showed that a 1 mg/m^3^ increase in CO concentrations was associated with a 4.4% increase in CVD hospital admissions [14]. However, the short-term effects of ambient CO exposure on cause-specific CVD hospitalizations in China remain unknown. Additionally, this association on the city-specific level has not comprehensively reported, especially in large cities, such as Beijing.

In this study, we aimed to examine the associations between ambient CO and total and cause-specific CVD hospital admissions in Beijing. We also investigated the robustness of this association after controlling for fine particulate matter, sulfur dioxide, nitrogen dioxide, and ozone. Stratified analyses by sex and age were further conducted to explore the associations in these susceptible subpopulations.

## Methods

### Health outcome data

**Fig. 1:**
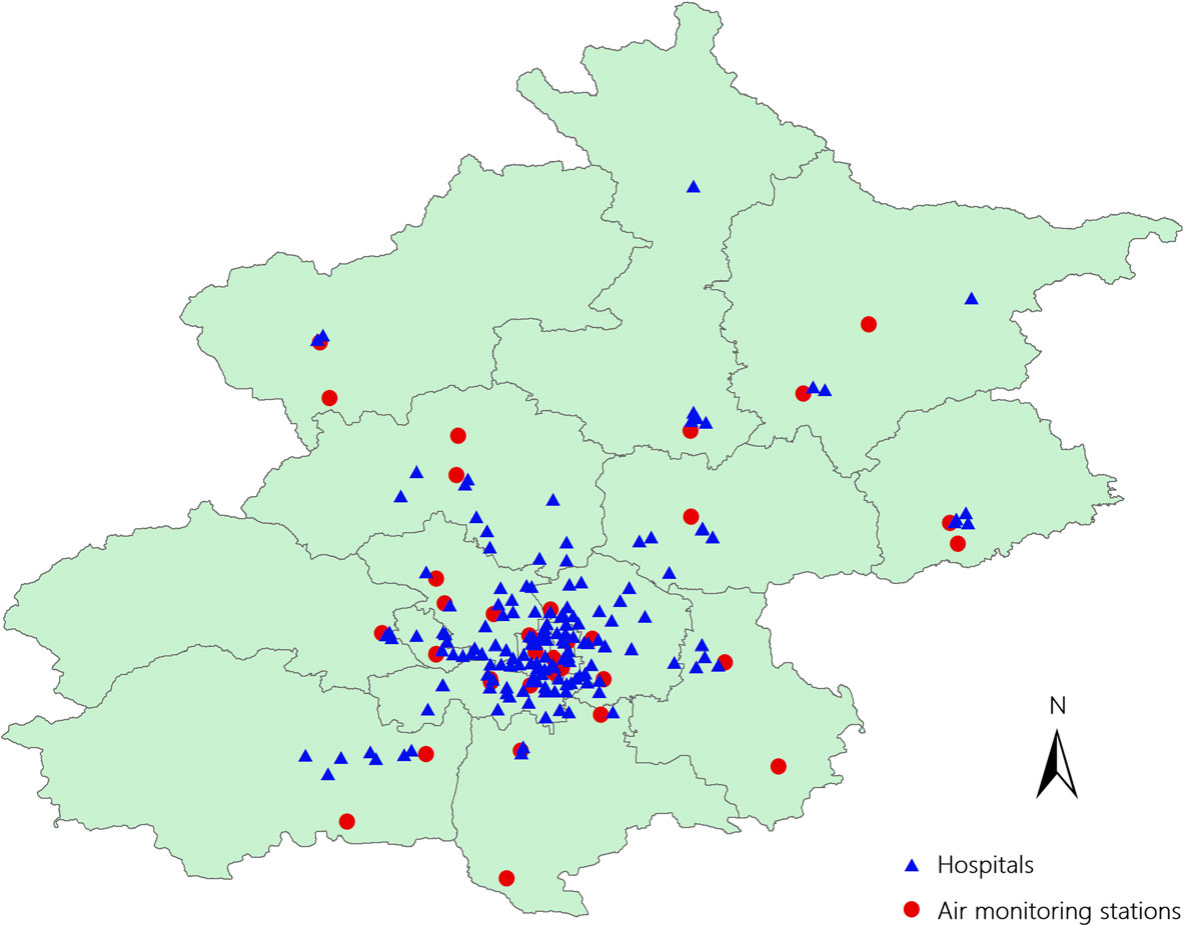
The locations of the air quality monitoring stations and hospitals

CVD hospital admission records were extracted from the Beijing Municipal Commission of Health and Family Planning Information Center (http://www.phic.org.cn/) between Jan 1, 2013, and Dec 31, 2017. The geographic locations of hospitals in this study are shown in Fig. 1. From each hospital admission record, we extracted information on the patient’s date of hospital admission, principal diagnosis, age, and sex. Cause-specific CVD hospitalizations were identified based on the primary diagnosis according to the following International Classification of Diseases, 10th Revision (ICD-10) codes: coronary heart disease (ICD-10: I20-I25, CHD), atrial fibrillation (ICD-10: I48, AF) and heart failure (ICD-10: I50, HF). In this study, total CVD admissions were calculated as the sum of CHD, AF and HF. Because few hospital admissions for CVD under the age of 18 years were recorded; thus, these individuals were excluded from the current analysis.

The Institutional Review Board at the Capital Medical University approved the study’s protocol (IRB00009511). Informed consent was waived as the data were typically used for administrative purposes. Only aggregated data was used for the current analysis.

### Air pollution and meteorological data

We obtained air pollution data between Jan 1, 2013, and Dec 31, 2017, from the Beijing Municipal Environmental Protection Bureau (http://www.bjepb.gov.cn/), which provided hourly monitoring data from 35 fixed-site air quality monitoring stations covering nearly every district (county level) in Beijing. The locations of monitoring stations are also shown in Fig. 1. The 24-h average concentrations of five pollutants, including carbon monoxide (CO), particulate matter less than 2.5 μm in aerodynamic diameter (PM_2.5_), sulfur dioxide (SO_2_), nitrogen dioxide (NO_2_) and daily maximum 8-h average ozone concentrations (O_3_) were used in this study. Moreover, to adjust for the potential confounding effects of weather conditions, meteorological data including the daily mean temperature, mean air pressure, and mean relative humidity were extracted for the same study period from the China Meteorological Data Sharing Service System (https://data.cma.cn/en).

### Study design

The case-crossover design [15, 16], which was first introduced by Maclure [17], is increasingly used in the literature to explore the short-term effects of air pollutant exposure on the risk of acute events [12, 18, 19]. In this study, a time-stratified case-crossover study design was used to compare each patient’s ambient CO exposure on the day of hospital admission for CVD (“case” day) to exposure on 3 or 4 reference days occurring on the same day of week in the same calendar month and year (“control” days). With this design, each stratum consisted of one case on the case day and several references on the control days; thus, biases due to long-term time trends and seasonal variations can be avoided [20]. Additionally, since individual patients serve as their own controls, individual characteristics (such as age, sex, marital status, education, etc.) are also well controlled [15].

### Statistical analysis

Conditional logistic regression is the standard statistical approach in the case-crossover analysis [21]. From statistical perspective, case-crossover design using a conditional logistic regression is a merely special case of time-series analysis [16, 22]. Typically, a stratum variable will be defined by including one case and all other referents as if a matched case-control study. Thus, if there are relatively large number of referents for each case, or if there are multiple cases on the same day, the stratum data must be expanded and repeated as many times in order to be modelled by conditional logistic regression, which is computationally inefficient. To minimize computational complications, we applied a conditional Poisson regression model which has been proven to give equivalent estimates as the conditional logistic model but allowing for over-dispersion and auto-correlation [23]. Conditional Poisson regression model is increasingly used for case-crossover analysis in environmental epidemiology [19, 23–25].

As mentioned previously, we first defined a stratum variable as a combination of the year, month and day of week, which was used to match a case and controls by the day of the week in the same calendar month. This variable can be used to control for time-dependent confounders (e.g., long-term trend, seasonality). Additionally, we controlled for weather conditions by including 4-day moving average of temperature (lag _0–3_), the same day of air pressure and relative humidity (lag _0_) as covariates for the model fit based on a prior study [18]. Considering that a nonlinear relationship had been shown between temperature, air pressure, relative humidity and hospitalizations for CVD in the previous studies [7, 19, 26], a natural cubic spline with three degrees of freedom (*df*) was used for each weather condition variable. Public holidays (except on Saturdays and Sundays) were regarded as a binary variable (yes or no) and adjusted in the model [4]. Thus, in the current analysis, the conditional Poisson regression model has the form:

$$ {Y}_t\sim Poisson\left({\mu}_t\right) $$

$$ Log\left({\mu}_t\right)=a+{\beta CO}_{\left(t,l\right)}+ ns\left({temperature}_{\left(t,l=0-3\right)}, df=3\right)+ ns\left({pressure}_{\left(t,l=0\right)}, df=3\right)+ ns\left( relative\_{humidity}_{\left(t,l=0\right)}, df=3\right)+\lambda {Stratum}_t+\upsilon {Holiday}_t $$

where *Y*_*t*_ is the observed daily CVD hospital admissions count on day t; *μ*_*t*_ is the expected daily CVD hospital admissions count on day t; *l* is the lag of the day; *β* is the coefficient for CO concentration on different lag days; *ns*(…) is the natural cubic spline; *df* is the degrees of freedom; *λ* is the coefficient for stratum that combines year, month and day of week; υ is the coefficient for public holidays.

We first conducted a single-pollutant model to estimate the linear effect of ambient CO on CVD hospital admissions using different lag patterns. The single-day effect of ambient CO was calculated on the same day (lag _0_) and up to the previous 5 days (from lag _1_ to lag _5_). The cumulative delay effect was calculated as the moving average of concentrations from the same day to the previous 1 days (lag _0–1_), to the previous 3 days (lag _0–3_) and to the previous 5 days (lag _0–5_). According to the previous studies [7, 10, 27], the 2-day moving average concentrations of CO (lag _0–1_) often produced the largest estimate effects on CVD morbidity and mortality [7]; therefore, we mainly reported the estimates effect at lag _0–1_. We further plotted exposure-response association curves using the nature cubic spline with three *df* for the concentrations of CO (lag _0–1_) [13]. The nonlinear association was examined by the Wald-test [28].

We also performed multipollutant models as sensitivity analyses to evaluate the robustness of our results. To remove the impact of collinearity between CO and other air pollutants (PM_2.5_, SO_2_, NO_2_, O_3_), principal component analysis (PCA) was used to establish the models [29]. We first substituted all original air pollutants by a composite latent variable (a linear combination of all air pollutants) through PCA, and then included this latent variable in conditional Poisson regression model. Meanwhile, the coefficient *β*^′^ of this latent variable was transformed into the coefficient *β* of the original air pollutants [30].

The potential effect modification on the association between ambient CO (lag _0–1_) and hospital admission for CVD was examined. First, subgroup analyses stratified by demographic characteristics (age group: 18–64 and ≥ 65 years; sex: male and female) were conducted in the single-pollutant model. A Z value was calculated to test the statistical significance of subgroups differences as follows: $\mathrm{Z}=\left({\beta}_1-{\beta}_2\right)/\sqrt{SE_1^2+{SE}_2^2}$, where *β*_1_ and *β*_2_ were the effect estimates for the two categories (e.g., male and female) and *SE*_1_ and *SE*_2_ were their respective corresponding standard errors [11, 31]. Finally, a *P* value was obtained from the standard normal distribution based on the Z value [31].

The relative risk (RR) was calculated from the conditional Poisson regression model (RR = *e*^*β*^). We reported the percentage changes (PC% = [RR-1] × 100%) in daily hospital admissions for CVD per 1 mg/m^3^ increase of CO concentrations and its corresponding 95% confidence interval (CI). All analyses were performed using Stata 14 (StataCorp., College Station, Texas), with the “xtpoisson” command to fit the conditional Poisson regression, “mkspline” command to build the nature cubic spline, “pca” command to conduct PAC, and “testparm” commend to perform the Wald-test. *P* value < 0.05 was deemed statistically significant.

## Results

We included 460,938 hospital admissions for CVD, including 378,090 CHD cases, 24,455 AF cases and 58,393 HF cases, during the 5-year study period in Beijing. Of those, 54.9% were males, and 37.6% were under 65 years of age. Table 1 shows the descriptive statistics of the daily hospital admissions (counts per day) for total and cause-specific CVD, air pollution concentrations, and weather conditions. On average, there were 252 hospital admissions for CVD per day, with a maximum of 749 admissions in a single day during the study period.

**Table 1:** Descriptive statistics for the daily hospital admissions for total and cause-specific cardiovascular disease, air pollution concentrations, and weather conditions in Beijing, 2013–2017

	Mean	SD	Minimum	P_2 5_	Median	P_7 5_	Maximum
Annual-average daily hospital admission							
Cardiovascular disease	252	165	9	106	192	405	749
Coronary heart disease	207	142	2	77	159	340	632
Atrial fibrillation	13	11	0	4	10	22	53
Heart failure	32	16	0	20	29	43	90
Annual-average CO concentrations, mg/m^3^							
	1.2	1.0	0.0	0.6	0.9	1.4	8.0
Annual-average pollution concentration (μg/m^3^)							
PM_2.5_	76.9	66.4	0.0	30.0	59.0	102.0	477.0
SO_2_	15.3	18.3	0.0	4.0	8.0	19.0	133.0
NO_2_	49.7	23.3	0.0	34.0	44.0	61.0	155.0
O_3_	95.7	63.0	0.0	49.0	80.8	136.0	366.7
Annual-average weather conditions							
Mean temperature, °C	13.9	10.9	−14.1	3.1	15.3	23.9	32.6
Relative humidity, %	24.4	26.7	1.1	5.2	7.5	43.4	95.3
Air pressure, hPa	1016.7	10.3	994.1	1008.0	1016.5	1025.3	1044.4

*P*_*25*_ 25th percentile, *P*_*75*_ 75th percentile, *CO* carbon monoxide, *PM*_*2.5*_ particulate matter with aerodynamic diameter less than 2.5 μm, *SO*_*2*_ sulfur dioxide, *NO*_*2*_ nitrogen dioxide, *O*_*3*_ ozone

The daily 24-h mean CO concentration in Beijing was 1.2 mg/m^3^ (SD: 1.0; range: 0.0–8.0). During the study period, the daily 24-h mean pollution concentration was 76.9 μg/m^3^ for PM_2.5_, 15.3 μg/m^3^ for SO_2_, and 49.7 μg/m^3^ for NO_2_, and the 8-h maximum mean concentration for O_3_ was 95.7 μg/m^3^. The daily mean ambient temperature was 13.9 °C, relative humidity was 24.4% and air pressure was 1016.7 hPa (Table 1).

The pairwise correlation among different air pollution concentrations and weather conditions during the study period is provided in Table 2. There were strong positive to moderate correlations between CO and PM_2.5_ (Pearson’s, *r* = 0.830), NO_2_ (*r* = 0.820) as well as SO_2_ (*r* = 0.670), but the correlation between CO and O_3_ was negative and weak (*r* = − 0.379). Additionally, the correlations between CO and each weather condition were relatively weak.

**Table 2:** Pearson correlation coefficients between air pollution concentrations and weather conditions in Beijing, 2013–2017

	CO	PM_2.5_	SO_2_	NO_2_	O_3_	Temperature	Relative humidity
CO	1.000						
PM_2.5_	0.830*	1.000					
SO_2_	0.670*	0.562*	1.000				
NO_2_	0.820*	0.786*	0.633*	1.000			
O_3_	−0.379*	− 0.149*	− 0.338*	− 0.383*	1.000		
Temperature	−0.398*	− 0.183*	− 0.488*	− 0.354*	0.768*	1.000	
Relative humidity	−0.028	−0.015	− 0.277*	− 0.017	0.029	0.135*	1.000
Air pressure	0.246*	0.061*	0.316*	0.221*	− 0.691*	− 0.875*	− 0.090*

* *P* < 0.001

*CO* carbon monoxide, *PM*_*2.5*_ particulate matter with aerodynamic diameter less than 2.5 μm, *SO*_*2*_ sulfur dioxide, *NO*_*2*_ nitrogen dioxide, *O*_*3*_ ozone

**Fig. 2:**
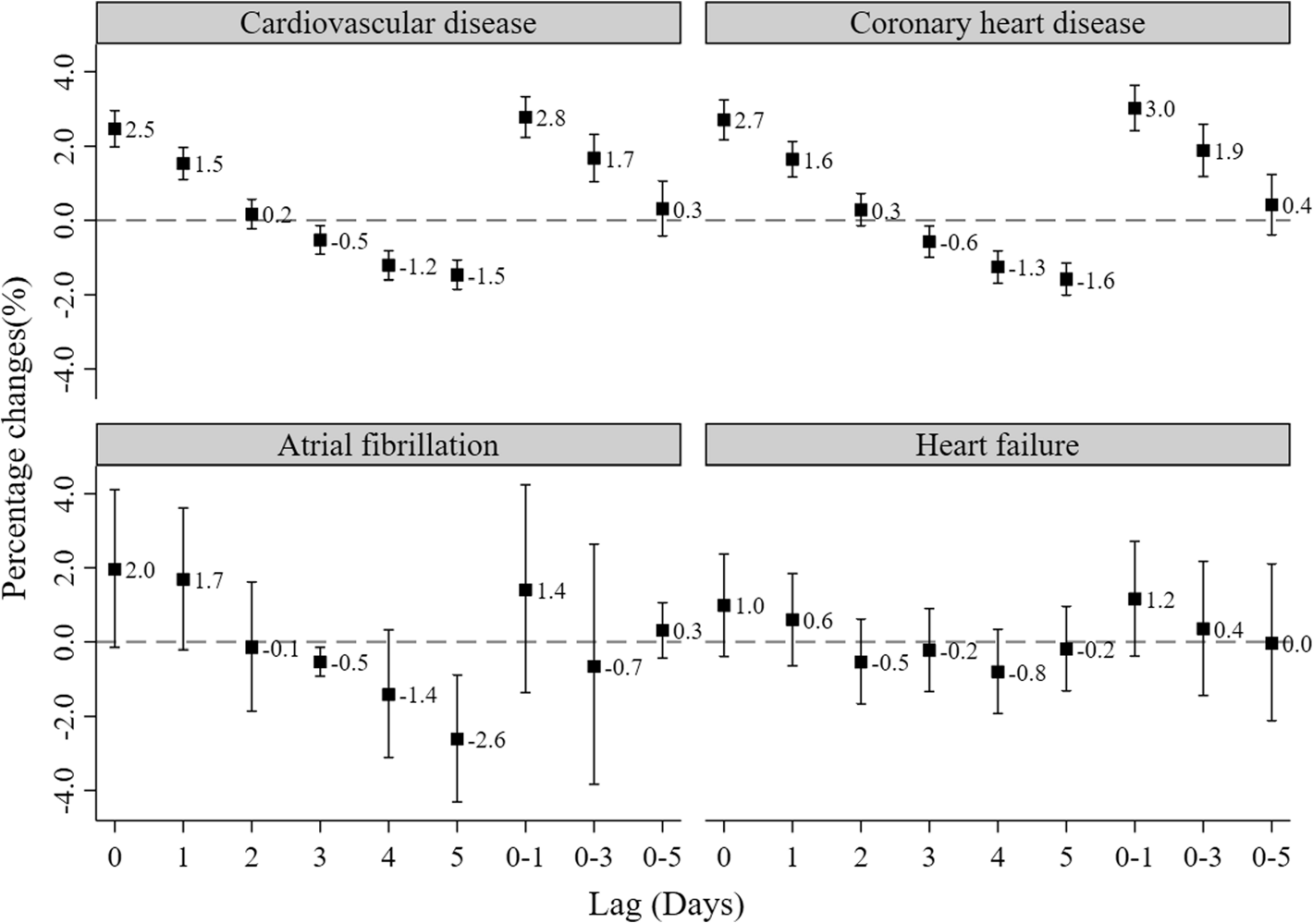
Percentage change with 95% confidence interval of hospital admissions for total and cause-special cardiovascular disease associated with a 1 mg/m^3^ increase in daily CO concentrations with varying lag patterns. Data are percentage changes (%) with 95% confidence intervals. Lag _0_ = current day. Lag _1_ = previous 1 day. Lag _2_ = previous 2 days. Lag _3_ = previous 3 days. Lag _4_ = previous 4 days. Lag _5_ = previous 5 days. Lag _0–1_ = 2-days moving average of lag _0_ - lag _1_. Lag _0–3_ = 4-days moving average of lag _0_ - lag _4_. Lag _0–5_ = 6-days moving average of lag _0_ - lag _5_

Fig. 2 shows the effects of CO exposure (1 mg/m^3^ increment) with varying lag patterns on the percentage change of daily hospital admission for CVD, CHD, AF and HF. The effect of ambient CO gradually decreased from lag _0_ to lag _5_ day. We found that ambient CO was positively and significantly associated with the percentage change in daily hospital admissions for CVD at lag _0_ and lag _1_ day, but negative and significant associations were observed at lag _4_ and lag _5_ day. The largest effect was observed on lag _0–1_ day. Therefore, the following analyses only focused on the cumulative delay effects of CO on lag _0–1_ day. The lag patterns for CHD were consistent with CVD. These associations between CO exposure and AF as well as HF, however, were not significantly detected on any lag day (all *P* > 0.05).

**Fig. 3:**
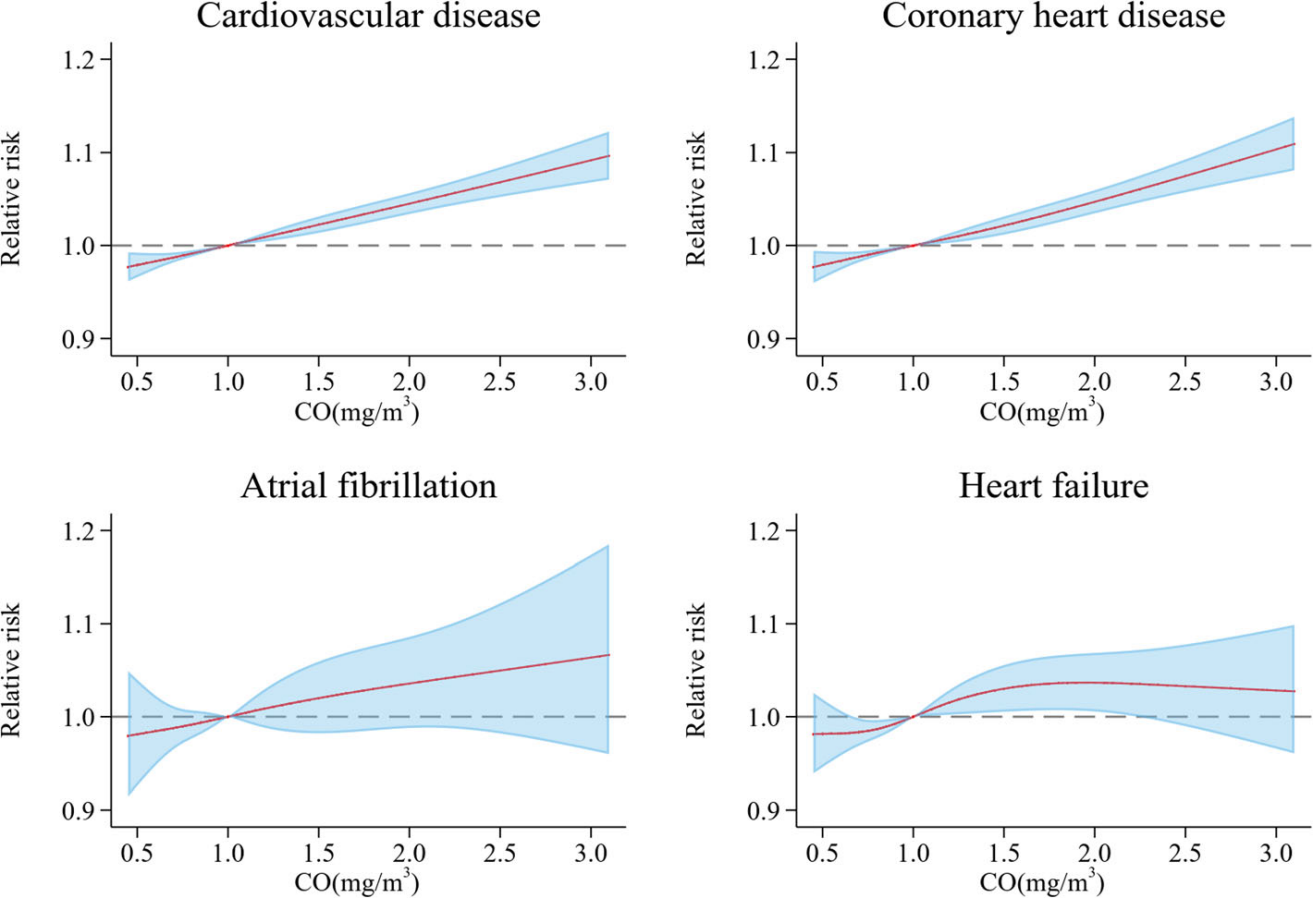
Exposure-response relationship curves for the association between hospital admissions for total and cause-special cardiovascular disease and the 2-day moving average (lag _0–1_) of carbon monoxide (CO) concentrations. The X-axis is the 2-day (lag_0–1_) moving average CO concentrations (mg/m^3^) truncated for the 5–95% percentiles of the distribution concentrations in the figure. The Y-axis is the relative risk (RR), after adjusting for temperature, relative humidity, air pressure, public holidays and long-term trends as well as seasonality. It is shown by the red solid line, and the blue shadow represent the 95% confidence interval

Figure 3 shows the exposure-response association curve between ambient CO concentrations on lag _0–1_ day and daily hospitalizations for total and cause-specific CVD. We did not find any apparent nonlinear exposure-response relationships for daily hospitalization for CVD (Wald χ^2^ = 0.01, *P*_for nonlinearity_ = 0.99), CHD (Wald χ^2^ = 0.61, *P*_for nonlinearity_ = 0.74), AF (Wald χ^2^ = 0.06, *P*_for nonlinearity_ = 0.97) or HF (Wald χ^2^ = 3.05, *P*_for nonlinearity_ = 0.21).

Table 3 shows the cumulative delay effects (lag _0–1_ day) of CO concentrations on daily hospital admissions for total and cause-specific CVD. In the single pollutant model, for a 1 mg/m^3^ increase in the 2-day moving average CO concentrations (lag _0–1_), we observed significant increases of 2.8% (95% CI: 2.2 to 3.3%) in the daily hospital admission for CVD, 3.0% (95% CI: 2.4 to 3.6%) for CHD, 1.4% (95% CI: -1.4 to 4.2%) for AF and 1.2% (− 0.4 to 2.7%) for HF. In the multipollutant models where copollutants were adjusted by PCA, the significant associations observed in the single-pollutant models weakened but remained significant for CVD and CHD. Additionally, a week association was observed between ambient CO and hospital admissions for AF.

**Table 3:** Percentage changes in daily hospital admission for total and cause-specific cardiovascular disease per 1 mg/m^3^ increase in 2-day moving average (lag _0 − 1_) concentration of carbon monoxide (CO), with and without adjustment of co-pollutants

	Percentage changes (%) and 95% confidence intervals	
	Single pollutant model ^a^	Multipollutant models ^b^
Cardiovascular disease	2.8 (2.2 to 3.3)	0.7 (0.5 to 0.9)
Coronary heart disease	3.0 (2.4 to 3.6)	0.8 (0.6 to 1.0)
Atrial fibrillation	1.4 (−1.4 to 4.2)	0.8 (0.1 to 1.5)
Heart failure	1.2 (−0.4 to 2.7)	0.2 (−0.3 to 0.7)

^a^Without adjustment of co-pollutants

^b^Adjustment of co-pollutants by principal component analysis

**Fig. 4:**
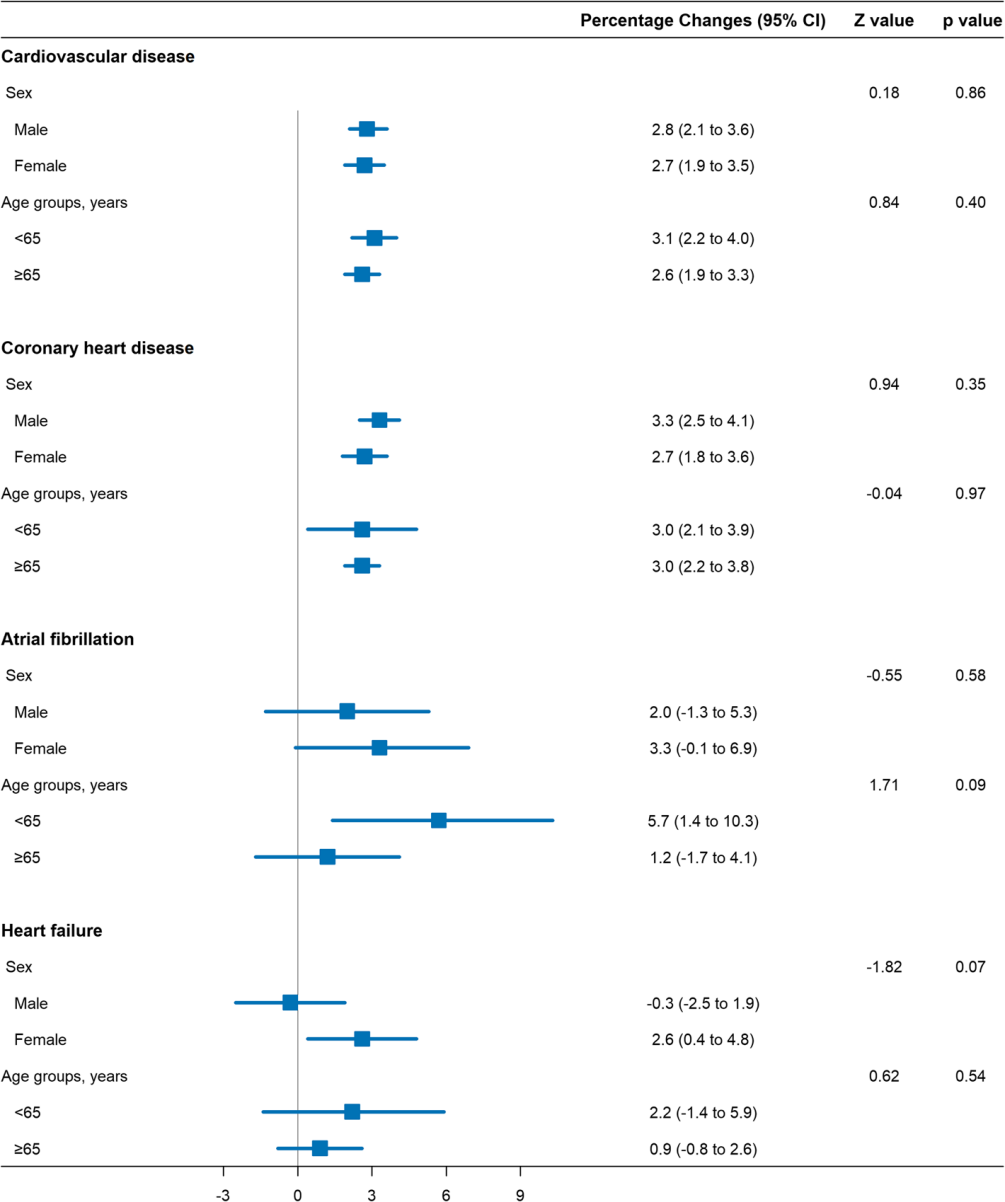
Percentage changes in daily hospital admission for total and cause-specific cardiovascular disease per 1 mg/m^3^ increase in 2-day moving average (lag _0–1_) concentration of carbon monoxide, stratified by sex (male and female) and age (< 65 and ≥ 65 years)

Figure 4 displays the associations between CO concentrations (lag _0–1_ day) and hospital admission for total and cause-specific CVD, stratified by age group, and sex. No effect modifications were found to be significant (all *P*_for heterogeneity_ > 0.05).

## Discussion

In the present study, we used a time-stratified case-crossover design with conditional Poisson model to investigate the associations of ambient CO and hospitalizations for CVD in Beijing from 2013 to 2017. We found that short-term exposure to ambient CO was significantly associated with an increased risk of hospital admissions for CVD and CHD, but not for AF and HF. The largest estimate effects were observed on the lag _0–1_ day. Our findings were robust after controlling for other pollutants in the multipollutant models.

Increasingly, scientific evidence has indicated that outdoor CO exposure is related to CVD morbidity and mortality [5–7, 10, 32–34]. For example, a multisite time-series study conducted by *Bell,* et al. found that a 1 mg/m^3^ increase in the same-day daily 1-h maximum CO was associated with a 1.0% (95% CI: 0.8 to 1.1%) increase in the risk of CVD admissions in 126 United States urban counties from 1999 to 2005 [10]. *Barnett,* et al. conducted a case-crossover study in seven Australian and New Zealand cities and combined the estimates across these cities using a random-effects meta-analysis. They found that a 0.9 mg/m^3^ increase in CO significantly increased the risk of hospital admissions for cardiovascular disease and ischemic heart disease by 2.2% (95% CI: 0.9 to 3.8%) and 2.3% (95% CI: 0.9 to 3.4%), respectively, in the elderly [33]. A study conducted in Arak, Iran reported that a 1 mg/m^3^ increase in CO concentrations on the same day was significantly associated with a 9.4% (95% CI: 5.1 to 14.0%) increase in total cardiovascular hospital admissions [5]. Additionally, the impact of ambient CO on cardiovascular outcomes was attenuated after controlling for other pollutants. For example, a study performed in Valencia, Spain showed that ambient CO was significantly related to emergency cardiovascular admissions in a single pollutant model, but this association was dismissed after adjusting for SO_2_ [32]. Similarly, in the *Bell,* et al*’* study, this association was attenuated after adjusting with same-day NO_2_ [10]. To reduce the collinearity between CO and other pollutants, principle component analysis was conducted in multipollutant models. PCA is a multivariate method and has been applied in various multipollutant studies. Our current findings were consistent with those of previous studies exploring the association between CO exposure and the risk of hospital admissions for CVD after adjusting for PM_2.5_, NO_2_, SO_2_ and O_3_ [7, 14].

In China, urbanization and economic development have greatly increased, especially in large cities such as Beijing and Shanghai. Gaseous air pollution (CO, SO_2_, O_3_, and NO_2_) has become a serious environmental problem. Ambient CO is primarily produced by incomplete combustion of carbon-containing fuels. The major source of CO in Beijing is generated from vehicle emissions. However, the adverse health effects of CO exposure on cardiovascular hospital admissions is understudied in China. Our findings were supported by a recent multicity analysis conducted in 14 large Chinese cities that reported a 0.8 mg/m^3^ increase in CO concentrations on lag _2_ day was significantly associated with a 1.1% (95% CI: 0.4 to 1.8%) increase in acute myocardial infarction admissions [35]. Moreover, associations between ambient CO and cardiovascular mortality had further been confirmed in a nationwide time-series analysis in 272 cities in China [7] and in the China Air Pollution and Health Effects Study (CAPES) [13]. Our results were consistent with previous studies performed in different Chinese cities [7, 13, 14, 35] that reported a significant CO exposure effect on lag _0_ and lag _1_ day, and a maximum CO exposure effect on lag _0–1_ day. Another recent study conducted in Beijing showed similar findings compared to our results [36]. Notably, a negative and significant association between CO exposure and hospitalizations for CVD or CHD was observed at lag _4_ and lag _5_ day, which indicated a presence of “harvesting effect” in the current analysis. Similarly, a time-series study conducted in Beijing in 2009–2010 also found that ambient CO had a negative effect on CVD mortality at lag _3_ and lag _4_ day [30], which was consistent with what we observed in the current study.

The exposure-response relationship was approximately linear and there was no noticeable threshold effect below which CO exposure had no effects on cardiovascular hospitalizations in the current analysis, which was in accordance with other findings in previous studies [7, 13]. However, a multicity study in the 26 largest cities in China showed a negative effect of CO exposure on total CVD admissions observed below 1 mg/m^3^ CO concentrations [14], which was also observed in our study. Therefore, further studies are necessary to explore the exposure-response relationship curve between CO levels and cardiovascular admissions.

We also found that the association between short-term CO exposure and hospital admissions varied by cause-specific CVD. The adverse effect was obvious and robust for daily hospitalizations for CHD, but not for AF or HF. Given the distinct differences in the underlying mechanisms through which that CO exposure triggers CHD compared with AF and HF [12, 37, 38], the impact of CO exposure on hospitalizations for cause-specific CVD could be substantially different. A previous meta-analysis that included only two prior studies suggested that CO pollutants were associated with an increased risk of AF [39]. The two prior studies used in the meta-analysis were conducted in the USA [40] and the UK [12]. The differences between China and these western countries in CO pollutants levels, weather conditions, and individual characteristics may partially explain the different adverse effects of ambient CO on hospitalization for AF. Additionally, a systematic review and meta-analysis with 35 high-quality studies demonstrated a 3.5% (95% CI: 2.5 to 4.5%) increase in hospitalizations or mortality for HF per 1 mg/m^3^ increment in CO concentrations [41]. However, none of those 35 studies included in the meta-analysis were conducted in China’s major cities [41]; thus the effects of CO pollution on hospitalizations for HF in China are less well- described [42]. Moreover, the prevalence of AF and HF in the Chinese population is considerably low. According to the China Multicenter Collaborative Study on Cardiovascular Epidemiology, the estimated prevalence of AF and HF in China is 1.0% [43] and 0.9% [44], respectively. In our current analysis, we observed a relatively small annual-average daily count for hospital admission due to AF and HF is very small (Mean: 13 for AF and 32 for HF). Thus, we might not have enough statistical power to elucidate a significant association between ambient CO and hospital admissions for AF or HF. Nevertheless, the exposure-response curves showed that high ambient CO exposure increased the risk of hospital admissions for AF and HF despite the statistical insignificance.

Identifying subpopulations who are the most susceptible is critically important for policy-maker to promote a targeted reduction in ambient CO [45]. A few studies suggested that females and elderly people were vulnerable to CO pollution-induced cardiovascular risk compared with males and younger people [11, 46–48]. However, we did not find any differences in these effects when stratified by sex and age, implying that there is no convincing evidence for susceptible subgroups for the adverse CO exposure effects in China. This was consistent with previous epidemiological studies conducted in China [7, 42, 49] and UK [12]. Further studies are still warranted to evaluate whether sex and age are effect modifiers in the association between ambient CO and cardiovascular outcomes.

As previously shown, inhaled CO can bind to hemoglobin and form carboxyhemoglobin which decreases the oxygen-carrying capacity of hemoglobin and causes cellular hypoxia [50]. However, the concentration of ambient CO is considered too low to induce the toxic effects of CO [51]. Several plausible biological mechanisms underlying the association between low concentrations of ambient CO with CVD have been proposed, including cardiac dysfunction [52, 53], systemic inflammation [54, 55], oxidative stress [56, 57], and thrombotic reaction [55]. Both animal models and population-based cohort studies have demonstrated the cardiotoxicity of CO [48, 52, 53]. A recent longitudinal study confirmed that high-sensitivity C-reactive protein increased with short-term exposure to CO among 61 patients with cardiovascular disease [55]. Fibrinogen and D-dimer significantly also increased with ambient CO exposure, and these biomarkers are related to thrombotic reactions [54, 55]. Overall, the scientific evidence supports that ambient CO exposure is associated with cardiovascular risk.

Our study had several strengths. First, cardiovascular hospital admission data were obtained from an established monitoring system in Beijing; thus, misclassifications in cardiovascular outcomes were less likely to occur. Second, we explored the potential association between ambient CO and cause-specific CVD admissions. However, the limitations of our study also need to be noted. Similar to other studies exploring the impact of air pollutants on health outcomes, we need to carefully interpret and infer the cause-effect relationship between carbon monoxide exposure and hospitalizations for CVD due to the ecological design of the present study. We also used ambient CO data aggregated from fixed-site air quality monitoring stations to represent individual-level exposure, so measurement bias may exist.

## Conclusions

Our findings have elucidated that short-term exposure to ambient CO significantly increased the risk of hospitalizations for total CVD, especially for CHD, in Beijing. Further study is necessary to identify the relationship between ambient CO and hospital admissions for AF and HF.

## Abbreviations

AF: Atrial fibrillation
CHD: Coronary heart disease
CO: Carbon monoxide
CVD: Cardiovascular disease
HF: Heart failure
NO_2_: Nitrogen dioxide
O_3_: Ozone
PC: Percentage changes
PM_2.5_: Particulate matter with aerodynamic diameter less than 2.5 μm
RR: Relative risk
SO_2_: Sulfur dioxide
